# ALA Inhibits ABA-induced Stomatal Closure via Reducing H_2_O_2_ and Ca^2+^ Levels in Guard Cells

**DOI:** 10.3389/fpls.2016.00482

**Published:** 2016-04-11

**Authors:** Yuyan An, Longbo Liu, Linghui Chen, Liangju Wang

**Affiliations:** College of Horticulture, Nanjing Agricultural UniversityNanjing, China

**Keywords:** abscisic acid (ABA), 5-aminolevulinic acid (ALA), calcium, hydrogen peroxide, stomatal opening, drought tolerance

## Abstract

5-Aminolevulinic acid (ALA), a newly proved natural plant growth regulator, is well known to improve plant photosynthesis under both normal and stressful conditions. However, its underlying mechanism remains largely unknown. Stomatal closure is one of the major limiting factors for photosynthesis and abscisic acid (ABA) is the most important hormone in provoking stomatal closing. Here, we showed that ALA significantly inhibited ABA-induced stomatal closure using wild-type and ALA-overproducing transgenic *Arabidopsis* (*YHem1*). We found that ALA decreased ABA-induced H_2_O_2_ and cytosolic Ca^2+^ accumulation in guard cells with stomatal bioassay, laser-scanning confocal microscopy and pharmacological methods. The inhibitory effect of ALA on ABA-induced stomatal closure was similar to that of AsA (an important reducing substrate for H_2_O_2_ removal), CAT (a H_2_O_2_-scavenging enzyme), DPI (an inhibitor of the H_2_O_2_-generating NADPH oxidase), EGTA (a Ca-chelating agent), and AlCl_3_ (an inhibitor of calcium channel). Furthermore, ALA inhibited exogenous H_2_O_2_- or Ca^2+^-induced stomatal closure. Taken together, we conclude that ALA inhibits ABA-induced stomatal closure via reducing H_2_O_2_, probably by scavenging, and Ca^2+^ levels in guard cells. Moreover, the inhibitive effect of ALA on ABA-induced stomatal closure was further confirmed in the whole plant. Finally, we demonstrated that ALA inhibits stomatal closing, but significantly improves plant drought tolerance. Our results provide valuable information for the promotion of plant production and development of a sustainable low-carbon society.

## Introduction

5-Aminolevulinic acid (ALA) is an essential precursor in tetrapyrrole biosynthesis in organisms, such as such as chlorophyll and heme in plants. Since 1998, hormonal activities of ALA have been found in plant tissue culture (Bindu and Vivekanandan, 1998). In recent 20 years, more research indicates that ALA is not only an important intermediate in biological metabolism, but also a vital plant growth regulator which regulates several key physiological processes such as promoting plant growth and increasing plant stress tolerance (Akram and Ashraf, 2013). One of ALA’s outstanding roles is improving plant photosynthesis and thereby increasing growth. And, it is worth emphasizing that ALA improves plant photosynthesis efficiency not only under normal conditions (Hotta et al., 1997), but also under various stresses, such as cold (Hotta et al., 1998), salt (Nishihara et al., 2003), low light (Wang et al., 2004), water deficit (Liu et al., 2011), heat (Zhang et al., 2012), and heavy metal stresses (Ali et al., 2013; Tian et al., 2014), suggesting its great application potential in agriculture and forestry. However, to date, the proposed mechanisms underlying ALA-promoted photosynthesis include only the following: (1) boosting light-harvesting capability by increasing chlorophyll content (Youssef and Awad, 2008), (2) improving photosynthetic electron transport activity (Wang et al., 2010), (3) promoting antioxidant activity (Nishihara et al., 2003), and (4) increasing rubisco activity by up-regulating transcription of gene encoding *Rubisco small unit* (Shen et al., 2011). Therefore, the mechanism how ALA regulates plant photosynthesis and growth is still in its infancy.

Except non-stomatal factors, stomatal behavior also plays important roles in plant photosynthesis. In fact, stomatal resistance is thought to be the major limiting factor for CO_2_ uptake by plants (Wang et al., 2014). And many reports have demonstrated that stomatal aperture is a limiting factor in photosynthesis and plant growth (Lawson and Blatt, 2014; Wang et al., 2014). Wang et al. (2004) firstly showed that exogenous ALA significantly increased stomatal conductance of melon (*Cucumis melo*) seedlings. Subsequently, several researchers reported that ALA could reduce stomatal limitation in date palm (*Phoenix dactylifera*) seedlings (Youssef and Awad, 2008), and enhance stomatal conductance in leaves of pepper (*Capsicum annuum*; Korkmaz et al., 2010), oilseed rape (*Brassica napus*; Naeem et al., 2010), ‘Summer Black’ grape (Xie et al., 2013a) and apple (Gao et al., 2013) seedlings. Based on the above findings, we assume that the promoting effect of ALA on stomatal aperture might be universal in plant, and ALA-induced stomatal opening should be a critical mechanism for improvement of plant photosynthesis. However, to our knowledge, no specific information is available regarding the regulatory effects of ALA on stomatal movement and its functional mechanisms.

Stomatal movement is a highly complex process and modulated by many stimuli. Abscisic acid (ABA) was considered as the most important regulatory signaling molecule (Dodd, 2003; Tanaka et al., 2005). ABA-induced stomatal movement is one of the best characterized signaling systems in plants. More than 20 components, including secondary metabolites and ion channels, have been shown to participate in ABA-induced stomatal closure (Li et al., 2006). Therefore, our research on ALA-induced stomatal movement is started with a question whether ALA influence ABA-induced stomatal closure. Hydrogen peroxide (H_2_O_2_) and Ca^2+^ are signaling molecules of widespread importance in plant responses to various biotic and abiotic stimuli, including pathogen challenge, drought stress, atmospheric pollutants, extremes of temperatures, gravitropism, hormones, cell development, and senescence (Neill et al., 2002; Tuteja and Mahajan, 2007). It has been demonstrated that increasing H_2_O_2_ production and the H_2_O_2_-activated elevation of cytosolic Ca^2+^ concentration ([Ca^2+^]_cyt_) in guard cells are important mechanisms for ABA-induced stomatal closing (Pei et al., 2000). Interestingly, exogenous applications of ALA could significantly decrease H_2_O_2_ content and increase activities of antioxidant enzymes including catalase (CAT), ascorbate peroxidase (APX), glutathione reductase (GR), and superoxide dismutase (SOD) in leaves of several plant species (Balestrasse et al., 2010; Korkmaz et al., 2010; Liu et al., 2011; Zhen et al., 2012). However, there is no information available on the effect of ALA on H_2_O_2_ content in guard cells. Based on the above clues, we hypothesized that ALA might inhibit ABA-induced stomatal closure by decreasing H_2_O_2_ accumulation and hence [Ca^2+^]_cyt_ in guard cells.

5-Aminolevulinic acid-overproducing transgenic *Arabidopsis* (*YHem1*) have been obtained by expressing yeast *Hem1* gene under the control of *Arabidopsis HemA1* promoter (Zhang et al., 2010). To test our hypothesis, here, we first investigated whether ALA inhibited ABA-induced stomatal closure. The results showed that both exogenous and *YHem1* expression inhibit ABA-induced stomatal closure. The mechanism behind ALA’s regulation of stomatal movement was then dissected, and the way ALA regulates stomatal aperture, through ALA itself or its metabolites such as chlorophyll, was discussed. Effect of ALA on drought tolerance of *Arabidopsis* was further evaluated to exclude the possibility of increase in plant sensitivity to drought stress by ALA-inhibited stomatal closure. Our results provide valuable information for understanding the function mechanisms of ALA and the promotion of plant production.

## Materials and Methods

### Plant Materials and Growth Conditions

*Arabidopsis* (*Arabidopsis thaliana*) of wild-type (Col-0) and ALA-over-producing transgenic lines (*YHem1*; Zhang et al., 2010) that derived from Col-0 background were used in this study. Seeds were surface sterilized with bleaching power (5%, w/v) for 20 min, washed with sterilized water three times, then germinated and grown on vermiculite. Seedlings were irrigated every other day with half-strength Hoagland nutrition, in a growth chamber at 23°C, a relative humidity of 60%, and under a PPFD of 150 μmol⋅m^-2^⋅s^-1^ in 8 h light/16 h dark cycles.

### Guard Cell Viability Test

Epidermal strips were pretreated for 2 h in opening buffer (50 mM KCl, 10 mM MES, and 0.1 mM CaCl_2_, pH 6.2) with different treatments. Strips were then incubated with 0.25 μM fluorescein diacetate (FDA) for 5 min. The guard cell viability was detected according to the method of Garcia-Mata and Lamattina (2010). Fluorescence pictures were obtained with a Nikon-TE300 digital camera coupled to a laser scanning confocal microscope (Leica TCS SP8 STED 3X, LSCM). Cell viability was quantified by counting the percentage of fluorescent guard cells relative to total guard cells in the bright field.

### Stomatal Bioassay

Stomatal bioassay was performed on abaxial epidermal strips which were peeled from the rosette leaves of 5–6-week-old plants 4 h after the beginning of the light period. Epidermal peels were floated, peeled-side down, on opening buffer and incubated under light conditions (PPFD 240 μmol m^-2^ s^-1^) for 2 h to open the stomata. For the application of ALA or various inhibitors, the epidermal peels with pre-opened stomata were transferred to the same buffer supplemented with 10 μM ABA (Sigma–Aldrich, St. Louis, MO, USA), with or without the addition of ALA (0.05–5 mg L^-1^), 1 mM LA (an inhibitor of ALA metabolism), 100 U mL^-1^ CAT (a hydrogen peroxide-scavenging enzyme), 100 μM AsA (an important reducing substrate for H_2_O_2_ removal), 10 μM DPI (an inhibitor of the H_2_O_2_-generating enzyme NADPH oxidase), 5 mM EGTA (a Ca-chelating agent), or 50 μM AlCl_3_ (an inhibitor of calcium channel) for a further 1 h under light conditions. Stomatal apertures were observed by a light microscope (Nikon TE100, 400×), using a fitted camera (MShot Digital Imaging System), and measured with a digital ruler in Adobe Photoshop 6.0 (Adobe systems, San Jose, CA, USA).

To avoid any potential rhythmic effects on stomatal aperture, experiments were always started at the same time of the day. In each treatment, 30 randomly selected apertures were scored and experiments were repeated three times. The data presented are means of 90 measurements ± SEs.

### Scanning Electron Microscopy

The rosette leaves of 5–6-week-old wild-type and ALA-over-producing transgenic plants were immersed in opening buffer (50 mM KCl, 10 mM MES, and 0.1 mM CaCl_2_, pH 6.2). For wild-type samples, three treatments were designed by applying 0.5 mg L^-1^ ALA, 10 μM ABA, 10 μM ABA and 0.5 mg L^-1^ ALA, respectively. And the opening buffer without ABA and ALA were set as control treatment. For transgenic samples (P_0_ and P_3_), leaves of each line were treated with or without 10 μM ABA. All samples were incubated under light conditions (PPFD 240 μmol m^-2^ S^-1^) for 2 h at 25°C, then rinsed with phosphate buffer (pH 7.2) and fixed in 2% glutaraldehyde for 1 h and 1% glutaraldehyde for another 7 h. Leaves were then rinsed with phosphate buffer (pH 7.2), and dehydrated in an ethanol series (30 to 50 to 60 to 70 to 80 to 90 to 97 to 2 × 100%). These fixed and dehydrated samples were critical point dried with CO_2_, sputter-coated with a thin layer of gold and photographed under a scanning electron microscopy (PHILIPS-XL30E SEM) at 500× magnification. Stomata were counted and stomatal apertures were measured with a digital ruler in Adobe Photoshop 6.0 (Adobe systems, San Jose, CA, USA).

### Determination of Endogenous ALA

Endogenous ALA content in *Arabidopsis* leaves was measured according to Harel and Klein (1972). Random 0.1 g leaves were homogenized in 200 mM acetic acid buffer (pH 4.6), and centrifuged at 5,000 ×*g* for 15 min. One milliliter of supernatant were added to 0.5 mL acetylacetone, and boiled for 10 min. After cooling, 0.5 mL Ehrlich’s reagent was added. The absorbance was recorded at 553 nm after static hierarchy for 7 min by spectrophotometer.

### Measurement of Endogenous H_2_O_2_ Using Confocal Laser-Scanning Microscopy

Endogenous H_2_O_2_ were measured with fluorescent indicator dye H_2_DCF-DA as described by He et al. (2011) with slight modifications. The epidermal strips, previously incubated for 4 h under conditions promoting stomatal opening, were placed into Tris-KCl buffer (10 mM Tris and 50 mM KCl, pH 7.2) containing H_2_DCF-DA (Sigma–Aldrich, USA) at 50 μM for 30 min, in the dark at 25°C. Excess dye was removed with fresh Tris-KCl buffer in the dark. Peels of wild-type *Arabidopsis* were then transferred to the opening buffer alone or opening buffer supplemented with 10 μM ABA (Sigma–Aldrich, USA), with or without the addition of ALA. And peels of *YHem1* transgenic plants were transferred to the opening buffer alone or opening buffer supplemented with 10 μM ABA. Peel fluorescence were observed 5 min later using a laser scanning confocal microscope (Leica TCS SP8 STED 3X, LSCM), with the following settings: ex = 488 nm, em = 525 ± 15 nm, power 10%, zoom 2, mild scanning, frame 512 × 512, and Time-course and Photoshop software.

### Determination of Intracellular Ca^2+^ Variations Using Confocal Laser-Scanning Microscopy

Intracellular Ca^2+^ variations were determined with fluorescent dye Fluo-3 AM (Dojindo, Japan). The epidermal strips, previously incubated for 4 h under conditions promoting stomatal opening, were placed into MES-KCl solution containing Fluo-3 AM (dissolved in DMSO, Sigma) at 1 μM for 2 h, in the dark at 4°C. Excess dye was removed with fresh MES-KCl buffer in the dark. Peels of wild-type *Arabidopsis* were then transferred to the opening buffer alone or opening buffer supplemented with 10 μM ABA (Sigma–Aldrich, St. Louis, MO, USA), with or without the addition of ALA. And peels of *YHem1* transgenic plants were transferred to the opening buffer alone or opening buffer supplemented with 10 μM ABA. Peel fluorescence were observed 5 min later using a laser scanning confocal microscope (Leica TCS SP8 STED 3X, LSCM), with the following settings: ex = 488 nm, em = 525 ± 15 nm, power 10%, zoom 2, mild scanning, frame 512 × 512, and Time-course and Photoshop software. In the determination of H_2_O_2_ and Ca^2+^, at least five biological replicates were performed and three images taken for each biological replicate.

### Treatment with ABA in Whole Plant

Treatments with ABA in the whole plant, including wild-type and *YHem1*-transgenic plants, were carried out by irrigation of 10 μM ABA dissolved in distilled water for 30 min in growth chamber. For wild-type plants, four treatments, i.e., control, ALA, ABA, and ABA together with ALA, were set to examine the effects of exogenous ALA on ABA-induced stomatal closure *in planta*. *YHem1*-transgenic plants were treated with or without ABA to examine the effects of endogenous ALA. For drought stress, leaves of 5-week-plants were detached from the treated plants and fresh weight (FW) were recorded immediately. Leaves were then placed in the growth chamber for another 1 h, and the reduced weight was measured at 10 min intervals. Ratios of reduced weights to the original FW were calculated to evaluate the rate of FW decrease.

### Drought Tolerance Assay

Wild-type and *YHem1* transgenic *Arabidopsis* seeds were surface sterilized with 75% alcohol for 45 s and 10% NaClO for 10 min, wished with sterilized water three times, then placed on MS medium in Petri dishes. Forty-nine seeds were placed in each Petri dish. All materials were incubated in growth chamber at 25°C under PPFD of 150 μmol m^-2^ S^-1^ with 12 h light/12 h dark cycle. Ten days later, wild-type plants were randomly divided into four groups and *YHem1* transgenic plants were divided into two groups for six treatments. Six milliliter sterilized water or 15% PEG 6 000 were added to each Petri dish with or without 0.5 mg L^-1^ ALA. Seedlings were allowed to grow in the growth chamber for another 14 days. Then, seedlings were photographed and collected for determination of shoot and root length, stomatal aperture, and leaf chlorophyll contents.

Stomatal apertures were immediately observed by a light microscope (Nikon TE100, 400×), using a fitted camera (MShot Digital Imaging System), and measured with a digital ruler in Adobe Photoshop 6.0 (Adobe systems, San Jose, CA, USA). Leaf chlorophyll were extracted by 95% ethanol and determined according to Lichtenthaler and Wellburn (1982).

### Statistical Analysis

All data were taken from at least three independent experiments. Statistical analysis was performed using SPSS statistical computer package (version 16.0 SPSS Inc., Chicago, IL, USA). Data was compared with the control or among treatments by analysis of variance (ANOVA) to discriminate significant differences at *P* < 0.05 or *P* < 0.01 followed by least significant difference tests (LSD).

## Results

### Exogenous ALA Inhibits ABA-induced Stomatal Closure

**FIGURE 1:**
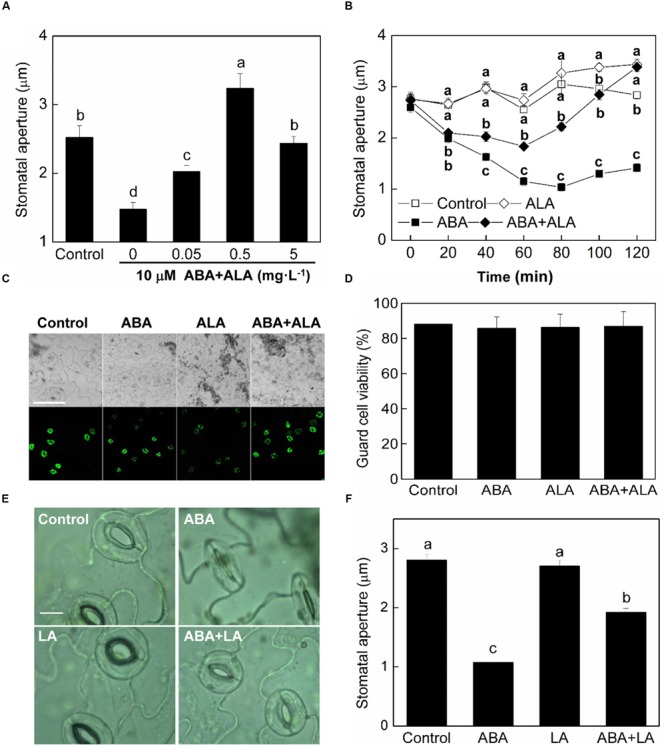
**ALA inhibits ABA-induced stomatal closure. (A)** Effects of different concentrations of ALA on ABA-induced stomatal closure. Isolated epidermal strips of wild-type *Arabidopsis* were incubated at 25°C in CO_2_-free MES-KCl buffer without ABA (Control), or containing 10 μM ABA and different concentrations of ALA under light (240 μmol m^-2^ s^-1^), and stomatal apertures were determined after 2 h. **(B)** Time courses of stomatal responses to 0.5 mg L^-1^ ALA, 10 μM ABA, or 10 μM ABA + 0.5 mg L^-1^ ALA, respectively. Different letters on the same time point indicate significant differences at *P* < 0.01. **(C,D)** Guard cell viability observed by fluorescence microscopy. Isolated epidermal strips of wild-type *Arabidopsis* were incubated at 25°C in either buffer (Control), or containing 10 μM ABA, 0.5 mg L^-1^ ALA, 10 μM ABA + 0.5 mg L^-1^ ALA for 2 h under light (240 μmol m^-2^ s^-1^), respectively, and then loaded with 0.25 μM fluorescein diacetate for 5 min. Images **(C)** obtained with a Nikon-TE300 digital camera depict one representative picture from three independent experiments. The upper image is in bright field and the lower image is in fluorescence. Scale bar: 100 μm. Cell viability **(D)** was quantified by counting the percentage of fluorescent guard cells relative to total guard cells in the bright field. **(E,F)** Levulinic acid (LA) inhibits ABA-induced stomatal closure. Isolated epidermal strips of wild-type *Arabidopsis* were incubated at 25°C in either buffer (Control), or containing 10 μM ABA, 1 mM LA (an analog of ALA which can block ALA metabolism), 10 μM ABA + 1 mM LA for 1 h under light (240 μmol m^-2^ s^-1^), respectively, and then images **(E)** were recorded and stomatal apertures **(F)** were determined. Scale bar: 10 μm. Values in **(A,B,F)** are the means of 90 measurements ± SE from three independent experiments. Different small letters represent significant differences among treatments (*P* < 0.01).

Abscisic acid is the most well-known elicitor of stomatal closure. To explore the regulatory mechanisms underlying ALA-induced stomatal opening, we examined the effects of ALA on ABA-induced stomatal closure. We employed an *in vitro* system using isolated epidermal peels in which we could measure stomatal apertures. In our experiments, after 2 h illumination of wild-type plants, the stomata opened and their apertures reached approximately 2.52 μm (**Figure 1A**). ABA application significantly reduced stomatal aperture to approximately 1.48 μm. When different concentrations of ALA were applied together with ABA to the isolated epidermal peels, ABA-induced stomatal closure was largely suppressed. The inhibitive effect of 0.5 mg⋅L^-1^ ALA was the most significant, with stomatal apertures increasing to approximately 3.24 μm which was even much higher than control. Therefore, 0.5 mg⋅L^-1^ was chosen as the final concentration of exogenous ALA for the following experiments. The time course for stomatal movement induced by ABA alone or ABA and ALA together illustrated that the inhibition of ALA on ABA-induced stomatal closure was initiated before 40 min and lasted for at least 2 h (**Figure 1B**). These observations indicated that exogenous ALA has an inhibitive effect on ABA-induced stomatal closure.

As no data are available in relation to ALA treatments of guard cells, to exclude that ALA could be toxic to guard cells, we detected that guard cell viability under ALA treatment. Epidermal peels were treated with 0.5 mg⋅L^-1^ ALA solution with or without 10 μM ABA and then loaded with 0.25 μM FDA. Result showed that guard cell viability was not influenced by ALA treatment (**Figures 1C,D**), indicating that ALA is not toxic for guard cells.

Levulinic acid (LA), an analog of ALA, is a competitive inhibitor of ALA dehydratase (ALAD) and has been used widely to block ALA metabolism, which leads to accumulation of endogenous ALA (Xie et al., 2013b). To confirm that it was ALA *per se* that was responsible for the inhibitive effect of ALA on ABA-induced stomatal closure, we applied LA instead of ALA. Following treatment with 10 mM LA, ABA-induced stomatal closure was also inhibited (**Figures 1E,F**). Thus, the inhibitive effect of ALA on ABA-induced stomatal closure probably resulted from ALA itself.

### Over-produced Endogenous ALA Inhibits ABA-induced Stomatal Closure

**FIGURE 2:**
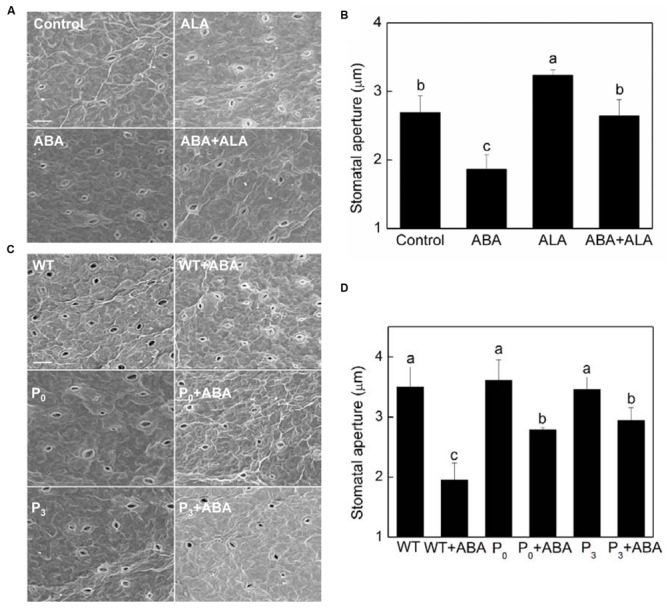
**Stomatal aperture observed by a scanning electron microscopy. (A,B)** Exogenous ALA inhibits ABA-induced stomatal closure. Wild-type *Arabidopsis* leaves were floated on either MES-KCl buffer (Control), or containing 10 μM ABA, 0.5 mg L^-1^ ALA, 10 μM ABA, and 0.5 mg L^-1^ ALA, respectively, at 25°C for 2 h, and then scanning electron microscopy (PHILIPS-XL30E SEM) images **(A)** of these leaves were recorded, based on which, stomatal apertures **(B)** were measured. **(C,D)** Over-produced endogenous ALA inhibits ABA-induced stomatal closure. Leaves of wild-type and *YHem1* transgenic (P_0_ and P_3_) *Arabidopsis* were floated on either CO_2_-free MES-KCl buffer alone or containing 10 μM ABA at 25°C for 2 h. Then scanning electron microscopy (PHILIPS-XL30E SEM) images **(C)** of these leaves were recorded, based on which, stomatal apertures **(D)** were measured. Scale bar: 25 μm. Values are the means of 90 measurements ± SE from three independent experiments. Different small letters represent significant difference between treatments (*P* < 0.01).

A similar pattern of changes in stomatal aperture was observed in exogenous ALA treated plants by scanning electron microscopy (**Figures 2A,B**), confirming the inhibitive effect of exogenous ALA on ABA-induced stomatal closure. To further evaluate the effects of ALA on ABA-induced stomatal closure, *YHem1*-transgenic *Arabidopsis* lines (P_0_ and P_3_) overproducing ALA were used. Stomatal responses of *YHem1*-transgenic lines and wild-type plants were compared using scanning electron microscopy. The result showed that the stomatal apertures of ALA-overproducing plants were similar to that of wild-type plants under normal condition, but were significantly larger under ABA treatment (**Figures 2C,D**). This result suggested that the over-production of endogenous ALA also inhibits ABA-induced stomatal closure.

Except stomatal aperture, stomatal development also has critical impact on plant photosynthetic capacity. To determine whether ALA influence plant stomatal development, we compared the stomatal density and size of ALA-overproducing transgenic plants to those of wild-type plants. The result showed that no significant differences were observed between them (**Table 1**), indicating that ALA does not affect stomatal development. Taken the results of exogenous ALA treatment together, we showed that ALA inhibits ABA-induced stomatal closure.

**Table 1:** Effect of ALA on stomatal density and size of *Arabidopsis*.

Plant	Stomatal density (number mm^-2^)	Stomatal length (μm)	Stomatal width (μm)
WT	288 ± 14^a^	20.91 ± 0.26^a^	17.55 ± 0.26^a^
P_0_	268 ± 19^a^	21.56 ± 0.24^a^	18.09 ± 0.24^a^
P_3_	305 ± 12^a^	21.35 ± 0.20^a^	18.47 ± 0.20^a^

*WT, wild-type; P_*0*_ and P_*3*_, two ALA-over-producing transgenic lines. Values are the means of 90 measurements ± SE from three independent experiments. The same letters within the same column indicate no significant difference at *P* = 0.05 level.*

### Levels of Endogenous ALA in WT and *YHem1*-transgenic *Arabidopsis* under Different Treatments

**FIGURE 3:**
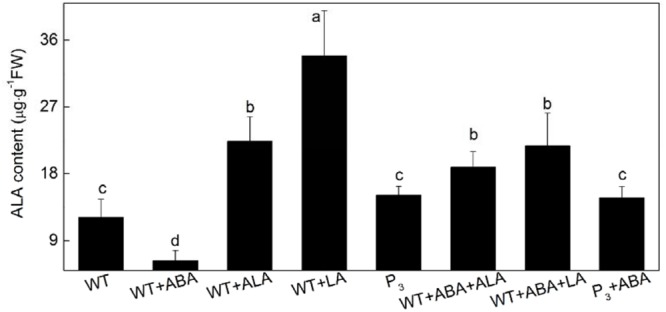
**Leaf endogenous ALA content.** Leaves of wild-type *Arabidopsis* were floated on either CO_2_-free MES-KCl buffer alone or containing 10 μM ABA, 0.5 mg L^-1^ ALA, 1 mM LA, 10 μM ABA + 0.5 mg L^-1^ ALA, 10 μM ABA + 1 mM LA at 25°C for 1 h under light (240 μmol m^-2^ s^-1^), respectively, and *YHem1* transgenic (P_3_) *Arabidopsis* were floated on either buffer alone or containing 10 μM ABA at 25°C for 1 h under light (240 μmol m^-2^ s^-1^). Then, leaves were collected and wished with distilled water for determination of endogenous ALA content. Values are the means of nine measurements ± SE from three independent experiments. Different small letters represent significant difference between treatments (*P* < 0.05).

To further confirm that it was the endogenous ALA that regulated guard cell ABA signaling, the effect of ABA on endogenous ALA level were determined during ABA-induced stomatal closure in the *YHem1* transgenic plants and ALA-, LA-treated wild-type plants. In wide-type plants, endogenous ALA content was significantly reduced by ABA alone treatment, but dramatically increased by exogenous ALA or LA treatment (**Figure 3**). When ABA was applied together with ALA or LA, endogenous ALA content in wild-type plants also significantly increased. Compared to ABA-treated wild-type plants, ABA-treated *YHem1* transgenic plants showed significantly higher level of endogenous ALA. These results indicated that endogenous ALA level increases during the inhibition of ABA-induced stomatal closure, confirming that endogenous ALA regulates guard cell ABA signaling.

### ALA Reduces H_2_O_2_ Content in Guard Cells

**FIGURE 4:**
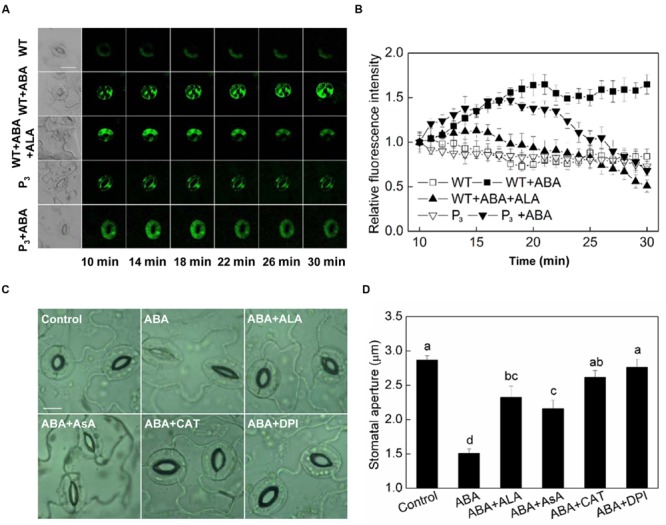
**ALA reduces ABA-induced H_2_O_2_ accumulation in guard cells. (A,B)** Changes in H_2_O_2_ content in guard cells. Isolated epidermal peels of pre-illuminated wild-type and *YHem1* transgenic (P_3_) *Arabidopsis* were loaded with H_2_DCF-DA for 30 min in darkness at 25°C, then excess dye was removed for the following treatments. Wild-type peels were transferred to either the opening buffer alone or supplemented with 10 μM ABA, 10 μM ABA + 0.5 mg L^-1^ ALA, and P_3_ peels were transferred to either the opening buffer alone or supplemented with 10 μM ABA. Five minutes later, fluorescence of the above-treated peels **(A)** was observed using a laser scanning confocal microscope (Leica TCS SP8 STED 3X, LSCM) and Time-course and Photoshop software. For each treatment, the first picture is bright field image and the following are fluorescence images corresponding to the bright field image at 1 min intervals. Scale bar: 20 μm. **(B)** Time course changes of the relative fluorescence in guard cells of each treatment. Data are normalized by calculating the relative changes in fluorescence over initial values. Values are the means of 15 measurements ± SE from three independent experiments. **(C,D)** The inhibitive effect of ALA on ABA-induced stomatal closure is similar to AsA, CAT, and DPI, all of which inhibit ABA-induced stomatal closure via decreasing H_2_O_2_ in guard cells. Isolated epidermal strips of wild-type *Arabidopsis* were incubated at 25°C in either buffer (Control), or containing 10 μM ABA, 10 μM ABA + 0.5 mg L^-1^ ALA, 10 μM ABA + 100 μM AsA, 10 μM ABA + 100 U mL^-1^ CAT, 10 μM ABA + 10 μM DPI for 1 h under light (240 μmol m^-2^ s^-1^), respectively, and then images **(C)** were recorded and stomatal apertures **(D)** were determined. Images **(C)** were recorded by light microscopy (Nikon TE100, 400×), using a fitted camera (MShot Digital Imaging System). Scale bar: 10 μm. Values are the means of 90 measurements ± SE from three independent experiments. The same letters represent no significant differences between treatments (*P* < 0.01).

Hydrogen peroxide (H_2_O_2_) is an important signaling molecule in guard cells (Neill et al., 2002), and its role in ABA-induced stomatal closure has been well established (Pei et al., 2000; Zhang et al., 2001a). To determine whether ALA inhibits ABA-induced stomatal closure via manipulating H_2_O_2_ content in guard cells, we first investigated the effect of ALA on H_2_O_2_ content in guard cells using a fluorescent dye, H_2_DCF-DA. We found that ABA increased H_2_O_2_ content in guard cells rapidly indicated by the increase in fluorescence intensity (**Figure 4A**). Significant H_2_O_2_ production was observed within 10 min after the application of ABA, and H_2_O_2_ content continuously increased with time. When ALA was applied together with ABA, ABA-induced H_2_O_2_ was largely impaired after 18 min and continuously weakened (**Figures 4A,B**). The transgenic plants were also used here to confirm ALA effect on ABA-induced H_2_O_2_. Similarly, the H_2_DCF-fluorescence in guard cell of transgenic plants was continuously weakened after 18 min as compared with the wild-type (**Figures 4A,B**) under ABA treatment. These results indicated that exogenous ALA and the over-produced endogenous ALA can both decrease ABA-induced H_2_O_2_ accumulation in guard cells.

AsA, CAT, and DPI are the most important reducing substrate for H_2_O_2_ removal, a H_2_O_2_-scavenging enzyme and an inhibitor of the ROS-generating enzyme, NADPH oxidase, respectively. To investigate the relationship between ALA-inhibited stomatal closure and the levels of H_2_O_2_ in guard cells, the ABA-treated epidermal strips were applied simultaneously with AsA, CAT, and DPI. Similar to ALA, AsA, CAT, and DPI all inhibited ABA-induced stomatal closure (**Figures 4C,D**). These results suggested that the inhibitive effect of ALA on ABA-induced stomatal closure is associated with a decrease of H_2_O_2_ levels in guard cells.

### ALA Reduces Cytosolic Ca^2+^ in Guard Cells

**FIGURE 5:**
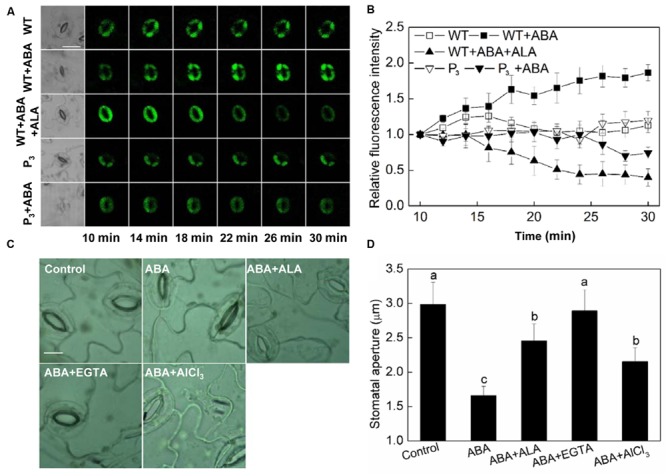
**ALA reduces ABA-induced cytosolic Ca^2+^ accumulation in guard cells. (A,B)** Changes in Ca^2+^ content in guard cells. Isolated epidermal peels of pre-illuminated wild-type and *YHem1* transgenic (P_3_) *Arabidopsis* were loaded with 1 μM Fluo-3 AM (dissolved in DMSO) for 2 h in darkness at 4°C, then excess dye was removed for the following treatments. Wild-type peels were transferred to either the opening buffer alone or supplemented with 10 μM ABA, 10 μM ABA + 0.5 mg L^-1^ ALA, and P_3_ peels were transferred to either the opening buffer alone or supplemented with 10 μM ABA. Five minutes later, fluorescence of the above-treated peels **(A)** was observed using a laser scanning confocal microscope (Leica TCS SP8 STED 3X, LSCM) and Time-course and Photoshop software. For each treatment, the first picture is bright field image and the following are fluorescence images corresponding to the bright field image at 2 min intervals. Scale bar: 20 μm. **(B)** Time courses changes of the relative fluorescence in guard cells of each treatment. Data are normalized by calculating the relative changes in fluorescence over initial values. Values are the means of 15 measurements ± SE from three independent experiments. **(C,D)** The inhibitive effect of ALA on ABA-induced stomatal closure is similar to EGTA and AlCl_3_, both of which inhibit ABA-induced stomatal closure via decreasing cytosolic Ca^2+^ in guard cells. Isolated epidermal strips of wild-type *Arabidopsis* were incubated at 25°C in either buffer (Control), or containing 10 μM ABA, 10 μM ABA + 0.5 mg L^-1^ ALA, 10 μM ABA + 5 mM EGTA, 10 μM ABA + 50 μM AlCl_3_ for 1 h under light (240 μmol m^-2^ s^-1^), respectively, and then images **(C)** were recorded and stomatal apertures **(D)** were determined. Images **(C)** were recorded by light microscopy (Nikon TE100, 400×), using a fitted camera (MShot Digital Imaging System). Scale bar: 10 μm. Values are the means of 90 measurements ± SE from three independent experiments. Different small letters represent significant difference between treatments (*P* < 0.01).

Calcium ion is another important second messenger. It has been reported that ABA-induced H_2_O_2_ accumulation and the H_2_O_2_-activated cytosolic Ca^2+^ increase are important mechanisms for ABA-induced stomatal closure (Pei et al., 2000; An et al., 2008). Since we have showed that H_2_O_2_ play a crucial role in ALA-inhibited ABA-induced stomatal closure, we assumed that Ca^2+^ signal may be also involved in the inhibitive process of ALA on ABA-induced stomatal closure. To test this hypothesis, we first investigated the effect of ALA on ABA-induced cytosolic Ca^2+^ accumulation. The results showed that both exogenous ALA and the over-produced endogenous ALA decreased ABA-induced cytosolic Ca^2+^ accumulation in guard cells after 14 min (**Figures 5A,B**). Then, we compared the effect of ALA with EGTA (a Ca^2+^ chelator) and AlCl_3_ (a blocker of Ca^2+^ channel) on ABA-induced stomatal closure. Similar to ALA, both EGTA and AlCl_3_ suppressed ABA-induced stomatal closure (**Figures 5C,D**). These results suggested that Ca^2+^ signal is also involved in ALA-inhibited stomatal closure.

### ALA Inhibits H_2_O_2_- and Ca^2+^-induced Stomatal Closure

**FIGURE 6:**
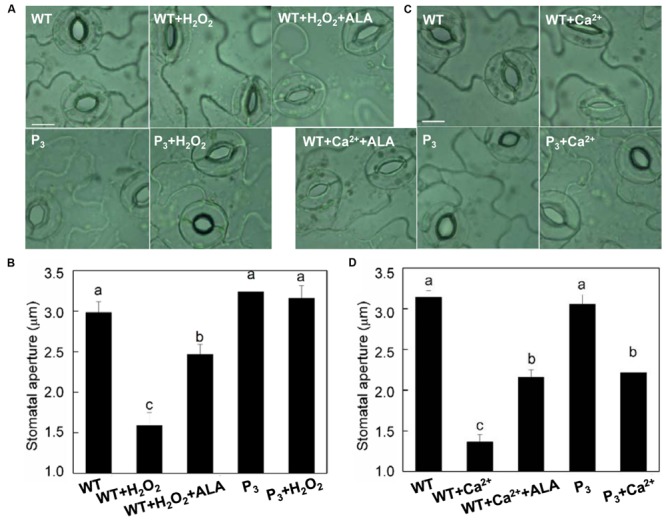
**ALA inhibits H_2_O_2_- and Ca^2+^-induced stomatal closures. (A,B)** ALA inhibits exogenous H_2_O_2_-induced stomatal closure. Isolated epidermal strips of wild-type *Arabidopsis* were incubated in either buffer alone or containing 200 μM H_2_O_2_, 200 μM H_2_O_2_ + 0.5 mg L^-1^ ALA, respectively, while P_3_ peels were incubated in either buffer or containing 200 μM H_2_O_2_ at 25°C under light (240 μmol m^-2^ s^-1^). One hour later, images **(A)** were recorded and stomatal apertures **(B)** were determined. **(C,D)** ALA inhibits exogenous Ca^2+^-induced stomatal closure. Isolated epidermal strips of wild-type *Arabidopsis* were incubated in either buffer alone or containing 2 mM CaCl_2_, 2 mM CaCl_2_ + 0.5 mg L^-1^ ALA, respectively, while P_3_ peels were incubated in either buffer or containing 2 mM CaCl_2_ at 25°C under light (240 μmol m^-2^ s^-1^). One hour later, images **(C)** were recorded and stomatal apertures **(D)** were determined. Images **(A,C)** were recorded by light microscopy (Nikon TE100, 400×), using a fitted camera (MShot Digital Imaging System). Scale bar: 10 μm. Values are the means of 90 measurements ± SE from three independent experiments. Different small letters represent significant difference between treatments (*P* < 0.01).

To further clarify whether ALA-induced stomatal movement by decreasing H_2_O_2_ content in guard cells, we first examined the effect of exogenous ALA on H_2_O_2_-induced stomatal closure in wild-type plants. H_2_O_2_ significantly reduced stomatal aperture (**Figures 6A,B**). However, when ALA was applied together with H_2_O_2_, H_2_O_2_-induced stomatal closure was largely repressed. We next compared the stomatal responses of wild-type plants with those of ALA-overproduced transgenic plants. Contrary to that of wild-type plants, stomatal aperture of transgenic plants was not reduced by H_2_O_2_ (**Figures 6A,B**). These observations indicated that both endogenous and exogenous ALA can scavenge H_2_O_2_, and then prevent stomatal closure induced by exogenous H_2_O_2_.

Similarly, both exogenous and endogenous ALA repressed Ca^2+^-induced stomatal closure significantly (**Figures 6C,D**), confirming that ALA inhibits ABA-induced stomatal closure by decreasing [Ca^2+^]_cyt_.

### ALA Inhibits ABA-induced Stomatal Closing in the Whole Plants

**FIGURE 7:**
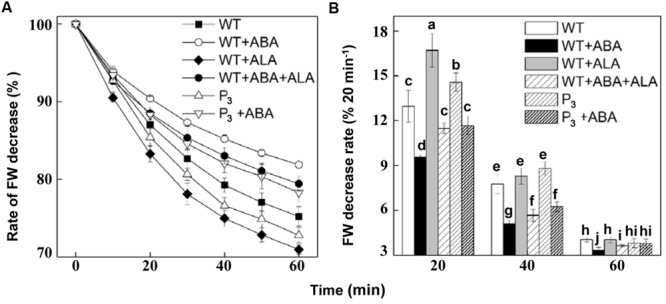
**ALA accelerates fresh weight (FW) decrease rate of plants. (A)** Changes in FW decrease ratio of detached leaves of wild-type plants, wild-type plants treated with 10 μM ABA, 0.5 mg L^-1^ ALA, or 10 μM ABA + 0.5 mg L^-1^ ALA, *YHem1* transgenic plants (P_3_) and *YHem1* transgenic plants treated with 10 μM ABA. **(B)** Leaf FW decrease rate calculated every 20 min after detachment from plants. FWs of the detached leaves were measured, and the ratios of reduced weights and original FWs were then calculated. Values are the means of four independent experiments, and five leaves were used for each treatment in one experiment. Error bars represent SEs. The same letters represent no significant differences between treatments (*P* < 0.05).

To verify the effect of ALA on stomatal movement in the whole plants, we next carried out an examination of whether ALA represses stomatal closure even *in planta*. Since stomata are known to close in response to drought to limit water loss by transpiration, we monitored time courses of leaf FW decrease after detachment from the whole plant. Results showed that ABA significantly reduced FW decrease rate, while exogenous ALA and expressing *YHem1* notably increased it, compared to that in non-treated wild-type plants (**Figure 7A**). When ABA was applied together with ALA to wild-type plants, the FW decrease rate in ABA-treated plants was obviously accelerated. Similarly, FW decrease rate in ABA-treated transgenic plants was faster than that in ABA-treated wild-type plants. The rate of FW decrease per 20 min also revealed that FW decrease rate in P_3_ and ALA-treated plants was higher than in non-treated plants within 40 min after detachment (**Figure 7B**). And FW decrease rate in ABA-treated wild-type plants was also higher in the presence of ALA, regardless of its source, during the whole experiment (**Figure 7B**). These results indicated that both exogenous and endogenous ALA accelerates plant transpirational water loss, reflecting the stomatal opening induced by ALA.

### ALA Improves Drought Tolerance of *Arabidopsis*

**FIGURE 8:**
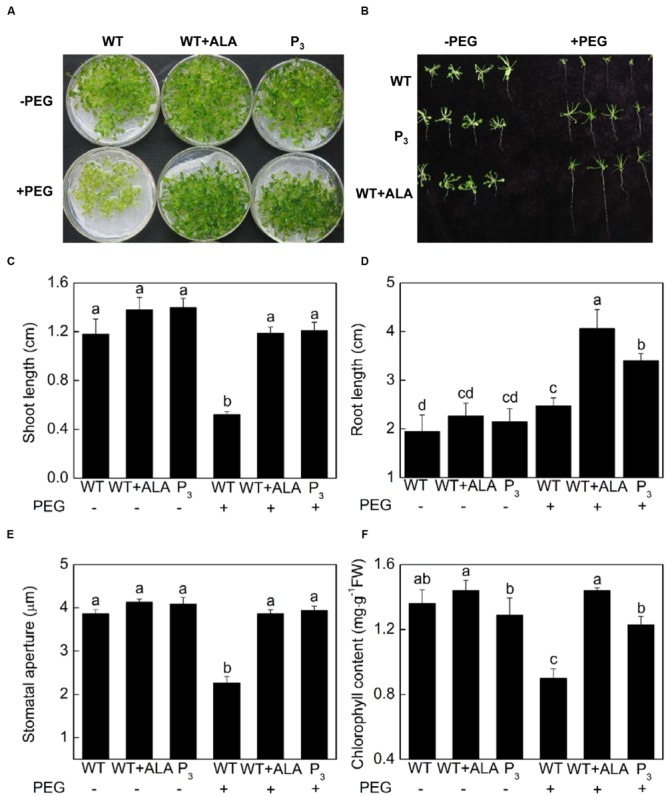
**ALA increases drought tolerance of *Arabidopsis*. (A,B)** ALA improves seedling growth under drought condition. 18-day-old wild-type *Arabidopsis* were cultured for another 14 days in either MS medium alone or containing 15% PEG 6 000 with or without 0.5 mg L^-1^ ALA. And *YHem1* transgenic (P_3_) *Arabidopsis* were cultured for another 14 days in MS medium alone or with 15% PEG 6 000. Then images were recorded. **(C,D)** ALA increases shoot length and root length of *Arabidopsis* seedlings exposed to drought condition. Values are the means of 18 measurements ± SE from three independent experiments. **(E)** ALA increases stomatal aperture of seedlings exposed to drought condition. Abaxial epidermal strips were peeled from leaves of the above treated plants and then stomatal apertures were determined. Values are the means of 90 measurements ± SE from three independent experiments. **(F)** ALA increases leaf chlorophyll content in seedlings exposed to drought condition. Values are the means of nine measurements ± SE from three independent experiments. The same letters represent no significant differences between treatments (*P* < 0.05).

Abscisic acid-induced stomatal closure is a well-known mechanism behind drought tolerance of plants. Since ALA inhibited ABA-induced stomatal closure, to determine whether ALA reduces plant drought tolerance, 15% PEG 6 000 were used to create drought stress, and ALA’s effect on growth characteristics, leaf chlorophyll content and stomatal aperture of *Arabidopsis* plants were investigated. Notably, the exogenous ALA-treated wild-type plants and the *YHem1* transgenic plants, which exhibited wider stomatal aperture, produced larger rosette leaves and longer roots, indicating they grew better than untreated wild-type plants under drought treatment (**Figures 8A–E**). Under 15% PEG 6 000, shoot length of the exogenous ALA-treated wild-type plants and the *YHem1* transgenic plants were 1.29 and 1.33 times, respectively, higher than that of the untreated wild-type plants, and root length were 0.64 and 0.37 times higher, respectively (**Figures 8B–D**). Under normal condition, no significant difference were found between chlorophyll content in untreated wild-type plants with *YHem1* transgenic plants or ALA-treated wild-type plants (**Figure 8F**). Drought stress significantly decreased chlorophyll content in untreated wild-type plants, but did not change that in *YHem1* transgenic plants or ALA-treated wild-type plants, resulting in significant higher level of chlorophyll in *YHem1* transgenic plants or ALA-treated wild-type plants than untreated wild-type plants. These results indicated that ALA-induced increase in stomatal aperture did not engender the sensitivity of plants to drought stress as expected. On the contrary, ALA significantly improved plant drought tolerance while increasing stomatal aperture. The significant positive correlation between chlorophyll content and stomatal aperture (*r* = 0.900; *P* = 0.014) in this experiment suggested that ALA-inhibited stomatal closure might be related to the improvement of chlorophyll synthesis.

## Discussion

### ALA Inhibits ABA-induced Stomatal Closure

5-Aminolevulinic acid can significantly improve leaf photosynthesis and widely promote plant growth and yield (Hotta et al., 1997, 1998; Nishihara et al., 2003; Wang et al., 2004; Liu et al., 2011). Stomata controls CO_2_ uptake for photosynthesis, determining plant productivity (Lawson and Blatt, 2014). Here, we showed that ALA does not affect stomata development, but significantly inhibits stomatal closure.

ABA is well-known in provoking stomatal closing (Aliniaeifard and van Meeteren, 2013; Kollist et al., 2014). In our study, exogenous ALA dramatically increased stomatal aperture under ABA treatment, indicating that ALA inhibits ABA-induced stomatal closure. This partially explains why exogenous ALA significantly improved plant photosynthesis even under stressful conditions (Hotta et al., 1997, 1998; Nishihara et al., 2003; Wang et al., 2004; Liu et al., 2011; Zhang et al., 2012). In addition, the stomatal aperture of P_0_ and P_3_, two lines of *YHem1*-transgenic plants (Zhang et al., 2010), were notably larger than that of wild-type plants under ABA treatment, indicating endogenous ALA also inhibits ABA-induced stomatal closure. This result further interprets why P_0_ and P_3_ grew more vigorously than wild-type plants which have been reported before (Zhang et al., 2010).

The effects of ALA on stomatal movement could be due to ALA itself or its metabolites. Wang et al. (2005) demonstrated that ALA-induced improvement of plant salt tolerance was dependent on its conversion into tetrapyrrole compounds. Similarly, Xie et al. (2013b) illustrated that ALA-induced promotion of anthocyanin accumulation in apple skins was not due to itself but its conversion to porphyrins. In the present study, however, LA showed similar inhibitive effect to ALA on ABA-induced stomatal closure. Since LA blocks ALA metabolism and hence leads to ALA accumulation (Xie et al., 2013b), the inhibitive effect of ALA on ABA-induced stomatal closure is probably due to ALA itself. The higher endogenous ALA contents in *YHem1*-transgenic plants and ALA-, LA-treated wild-type plants under ABA treatment confirmed that ALA itself participates in guard cell signaling. However, the significantly higher endogenous ALA but lower stomatal aperture in plants treated with ABA and LA than that in control plants suggested that ALA’s metabolites might also involve in regulating stomatal movement. ALA is the first key precursor of chlorophyll synthesis, and studies have demonstrated that exogenous ALA and *YHem1* expression significantly increase chlorophyll content in plants under stresses (Akram and Ashraf, 2013). Similarly, in this study, ALA increased chlorophyll content in *Arabidopsis* under osmotic stress (**Figure 8E**). A correlation has been previously suggested between chlorophyll synthesis and stomatal movement (Shimshi, 1967; Matsumoto et al., 2005). These studies showed that stomatal conductance was dependent on chlorophyll content to some extent. In the present study, stomatal apertures were significantly positively correlated with leaf chlorophyll content. These results indicate that ALA-improved chlorophyll synthesis may be involved in ALA-inhibited stomatal closure. But further studies are needed to elucidate whether and how ALA’s metabolites induce stomatal opening.

### ALA Inhibits ABA-induced Stomatal Closure through Reducing H_2_O_2_ and Ca^2+^ Levels in Guard Cells

Inhibition of ABA-induced stomatal closure results from either degradation of ABA or impairment of ABA signal. We did not measure ABA amount in our experiments, but ALA seems not to interfere with the early ABA-signaling pathway since the stomata started to close by ABA application even in the presence of ALA (**Figure 1B**). The stomata were kept half-opened or reopened by ALA treatment, indicating that ALA probably functions in some later stage of ABA-signaling. The increase in hydrogen peroxide (H_2_O_2_) production and the H_2_O_2_-activated elevation of [Ca^2+^]_cyt_ in guard cells are important downstream signaling components for ABA-induced stomatal closure (Pei et al., 2000). Previous studies have showed that ALA could decrease H_2_O_2_ levels in leaves or/and roots of several plant species (Balestrasse et al., 2010; Liu et al., 2011). However, little was known about the effect of ALA on H_2_O_2_ level in guard cells. In the present study, we showed that both exogenous and endogenous ALA decreased ABA-induced H_2_O_2_ accumulation in guard cells. The similarity of ALA’s effect to AsA, CAT, and DPI on ABA-induced stomatal closure further indicate that the inhibitive effect of ALA on ABA-induced stomatal closure results from the decrease of H_2_O_2_ levels in guard cells (Zhang et al., 2001a,b; Song et al., 2006; Wei et al., 2014).

Both cytokinins and auxins have been reported to induce stomatal opening by decreasing H_2_O_2_ levels in guard cells (Song et al., 2006). But the former probably initiates H_2_O_2_-scavenging systems, while the later mainly limits the production of H_2_O_2_ (Song et al., 2006). In the present study, both exogenous and endogenous ALA inhibited exogenous H_2_O_2_-induced stomatal closure, indicating that, similar to cytokinins, ALA reduced H_2_O_2_ levels probably by scavenging H_2_O_2_. Furthermore, ABA-induced H_2_DCF-fluorescence in our experiment first raised and then dropped after 18 min upon ALA application or in *YHem1*-transgenic plants (**Figure 4**), suggesting that H_2_O_2_ generated first and then was scavenged. This result confirmed that ALA reduced H_2_O_2_ levels mainly through accelerating its elimination. Until now, little is known about how ALA scavenge H_2_O_2_ in guard cells. However, many reports have revealed that ALA enhances plant antioxidant capacity in leaves or/roots. For example, ALA significantly improved ratio of GSH/GSSG and AsA/DHA and enhanced activities of several antioxidant enzymes including CAT, APX, and GR in oilseed rape (*Brassica napus*) under water-deficit stress (Liu et al., 2011). Similarly, ALA markedly increased APX, GR, and CAT activity and up-regulated the expressions of *CAT, cAPX*, and *GR* gene in NaCl-treated cucumber plants (Zhen et al., 2012). Therefore, in guard cells, ALA may also activate the antioxidant defense system to reduce H_2_O_2_ content. But this speculation and the specific antioxidant mechanisms in guard cells needs further testing.

Cytosolic Ca^2+^ in guard cells plays pivotal roles in stomatal function (Harada and Shimazaki, 2009). H_2_O_2_ activation of plasma membrane Ca^2+^ channel in guard cells is known as an important downstream component in ABA signaling (Pei et al., 2000). In the present study, ALA decreased ABA-induced [Ca^2+^]_cyt_ in guard cells, and ALA’s effect on ABA-induced stomatal closure was similar to EGTA, a Ca^2+^ chelator (Schwartz, 1985), and AlCl_3_, a blocker of Ca^2+^ channel (Zhao et al., 2007). In addition, ALA significantly inhibited Ca^2+^-induced stomatal closure. These results indicate that ALA inhibits ABA-induced stomatal closure by decreasing [Ca^2+^]_cyt_. As ABA-induced H_2_O_2_ accumulation was inhibited, the decrease of [Ca^2+^]_cyt_ might be a result of the suppressed Ca^2+^ channel activity. Besides, decrease of [Ca^2+^]_cyt_ start in 14 min after treatment (**Figure 5**), while reduction of H_2_O_2_ content begins after 18 min (**Figure 4**), suggesting that the time for [Ca^2+^]_cyt_ began to decrease was ahead of the reduction of H_2_O_2_ content. These results indicate that there are some H_2_O_2_-independent signal pathways leading to the decrease of [Ca^2+^]_cyt_ under ALA treatment. P-type Ca^2+^-ATPase can remove Ca^2+^ from the cytosol and hence reduce [Ca^2+^]_cyt_, and play an important role in Ca^2+^ signal (Kollist et al., 2014). Recently, We found that ALA increased Ca^2+^-ATPase activity, decreasing [Ca^2+^]_cyt_ in pollen tube of pear (*Pyrus pyrifolia*), and hence reduced the growth of pollen tube (An et al., 2016). Therefore, maybe this mechanism is also employed by ALA to regulate [Ca^2+^]_cyt_ in guard cells.

The above results demonstrate that ALA inhibits ABA-induced stomatal closure via reducing H_2_O_2_ and Ca^2+^ levels in guard cells, and the mechanisms on how ALA decrease H_2_O_2_ and Ca^2+^ levels in guard cells need to be further studied.

### ALA Improves Drought Tolerance while Increasing Stomatal Aperture

In the whole plant, upon drought stress, the rate of transpiration was greater in *YHem1* and wild-type plants exposed to ALA than in untreated wild-type control plants, indicating the inhibitive effects of ALA on ABA-induced stomatal closure were also observed *in planta*. However, inhibition of transpirational water loss by ABA- or stress-induced stomatal closing has been generally accepted as an important mechanism improving stress tolerance, especially the drought tolerance (Li et al., 2006). Whether ALA impairs plant drought tolerance? Here, we showed that 0.5 mg⋅L^-1^ exogenous ALA and *YHem1* expression, both of which inhibit ABA-induced stomatal closure, significantly improved drought tolerance of *Arabidopsis* (**Figure 8**). These results indicate that ALA-inhibited stomatal closure does not increase plant sensitivity to drought stress.

Actually, there have been a few studies about the effects of ALA on plant drought tolerance, and they proved that ALA improves drought tolerance of various plants, including wheat (*Triticum aestivum vulgare* L.; Al-Thabet, 2006; Kosar et al., 2015), barley (*Hordeum vulgare* L.; Al-Khateeb, 2006), oilseed rape (*Brassica napus* L.; Liu et al., 2011, 2013), and cucumber (*Cucumis sativus* L.; Li et al., 2011). Chlorophyll accumulation, improvement of photosynthetic electron transfer ability, photosynthesis, and antioxidant capacity play important roles in ALA-conferred drought tolerance (Li et al., 2011; Liu et al., 2011, 2013). Recently, Kosar et al. (2015) found that ALA significantly improved leaf glycine betaine accumulation and root K^+^ content under drought stress, indicating osmotic regulation also contributes to the improvement of drought tolerance by ALA. In addition, ALA significantly improved root dry weight under drought stress (Kosar et al., 2015), indicating ALA enhances plant root growth. Consistently, our data showed that both 0.5 mg⋅L^-1^ exogenous ALA and *YHem1* expression significantly improved root length (**Figures 8B,D**) of *Arabidopsis* under drought stress. Root system plays a critical role in plant adaptation to drought environments, in terms of signal perception and transmission, water and nutrient uptake during both drought and rewetting conditions (Sharp et al., 2003; Schachtman and Goodger, 2008). Therefore, improved root growth is an important mechanism behind ALA’s improvement of drought tolerance. In addition, aquaporins are known to significantly contribute to water movement and hence play important roles in plant drought tolerance (Zhao et al., 2015). Zhao et al. (2015) found that ALA controls aquaporin expression and consequently regulates plant water homeostasis under salt stress. Therefore, various mechanisms are involved in ALA-conferred drought tolerance, and the stronger root water uptake and aquaporin regulation maybe offset the adverse effect of ALA-induced stomatal opening on water loss. However, how ALA improves plant drought tolerance, especially the molecular mechanisms behind the paradox between enhanced drought tolerance and promoted stomatal opening, needs to be further studied.

### Physiological Role of ALA Inhibition of ABA-induced Stomatal Closure

Increasing reports indicate that stomatal resistance is an important limiting factor in photosynthesis and plant growth (Yang et al., 2012; Campos et al., 2014; Wang et al., 2014). However, to date, few studies have been carried out to promote stomatal opening with the goal of improving photosynthesis, perhaps because of the difficulty in balancing the counteracting effects of taking up CO_2_ while losing water through the stomata. Recently, there were some attempts that trying to improve photosynthesis by manipulating stomatal opening. However, the plant sensitivity to stresses was simultaneously increased in most cases. For example, *slac1*, an open-stomata mutant of rice, have been shown to increase leaf photosynthesis rate under well-watered conditions (Kusumi et al., 2012). But the *slac1* mutation had no effect on plant growth due to an increased sensitivity to drought stress. Therefore, efficient ways balancing plant stomatal opening and stress resistance are of urgent need in the present era of global climate changes and the threat of food insufficiency. In the present study, we demonstrate the inhibitive effect of ALA on stomatal closing and the concurrent improvement of plant drought tolerance, suggesting great application potential of ALA in agriculture and forestry.

Except drought stress, it has been well documented that low concentrations of ALA could markedly improve plant resistance to many other stressful conditions, including cold (Hotta et al., 1998), salt (Nishihara et al., 2003), low light (Wang et al., 2004), heat (Zhang et al., 2012), and heavy metal stress (Ali et al., 2013; Tian et al., 2014). And, in all these studies, improving photosynthesis and growth was proved to play important roles in ALA-increased stress resistance. In addition to the effect of exogenous ALA in these studies, expressing *YHem1* in *Arabidopsis*, which over-produced endogenous ALA, enhanced plant salt tolerance and growth as well (Zhang et al., 2010). Thus, it seems feasible to promote plant photosynthesis and improve plant resistance simultaneously to many abiotic stresses by application of ALA or through genetic modification of ALA biosynthesis.

In summary, the data presented here showed that ALA inhibits ABA-induced stomatal closure via reducing H_2_O_2_ and Ca^2+^ levels in guard cells, and simultaneously improves plant drought tolerance. Since ALA is naturally present in all living cells and has been proved to be of no toxicity and no pollution (Akram and Ashraf, 2013), application of ALA or expressing *YHem1* in crops and fuel plants is expected to contribute greatly to the promotion of plant production and a sustainable low-carbon society, especially under stressful conditions. The interaction between ALA and ABA in regulating stomatal movement would no doubt provide clues directing further study about functional mechanisms of ALA.

## Author Contributions

YA, LW conceived and designed research. LL and LC carried out all the experiments. YA, LL, and LC analyzed the data. YA and LW wrote the manuscript. All authors read and approved the manuscript.

## Conflict of Interest Statement

The authors declare that the research was conducted in the absence of any commercial or financial relationships that could be construed as a potential conflict of interest.
